# Water dispersible surface-functionalized platinum/carbon nanorattles for size-selective catalysis†
†Electronic supplementary information (ESI) available: General experimental details, Elemental analysis results, XRD, ICP-TOFMS, TG, electron microscopy, EDX and particle size distributions. See DOI: 10.1039/c7sc03785f


**DOI:** 10.1039/c7sc03785f

**Published:** 2017-10-30

**Authors:** Corinne J. Hofer, Robert N. Grass, Elia M. Schneider, Lyndsey Hendriks, Antoine F. Herzog, Martin Zeltner, Detlef Günther, Wendelin J. Stark

**Affiliations:** a Institute for Chemical and Bioengineering , ETH Zurich , Vladimir-Prelog-Weg 1 , 8093 Zurich , Switzerland . Email: wendelin.stark@chem.ethz.ch; b Laboratory of Inorganic Chemistry , ETH Zurich , Vladimir-Prelog-Weg 1 , 8093 Zurich , Switzerland

## Abstract

Selective dealloying of metal nanoparticles results in rattle-type hollow carbon nanoshells enclosing platinum nanoparticles, which are able to perform size-selective catalysis. Selective functionalization of the outer graphene-like carbon surface prevents agglomeration and leads to well dispersible nanocatalysts in aqueous solutions. The synthesis starts with the production of nanoparticles with a cobalt–platinum-alloy core surrounded by graphene-like carbon *via* reducing flame spray synthesis. After surface functionalization, simultaneous pore formation in the shell-wall and dissolution of the cobalt results in platinum encapsulated in hollow carbon nanospheres. Catalytic oxidation of differently sized sugars (glucose and maltoheptaose) reveales size-selective catalytic properties of these platinum nanorattles.

## Abstract 2

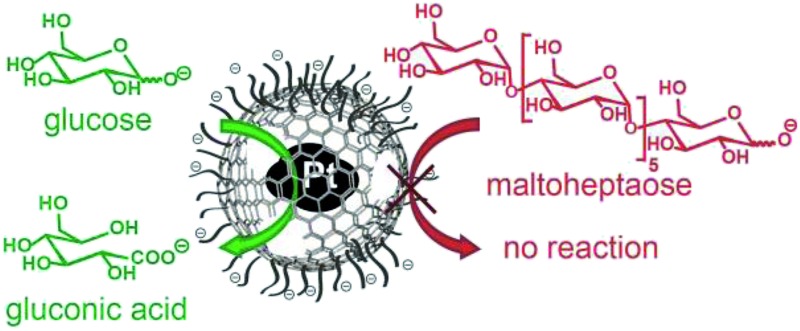
Surface-functionalized hollow carbon nanobubbles containing platinum in their interior perform size-selective catalysis.

## Introduction

**Fig. 1:**
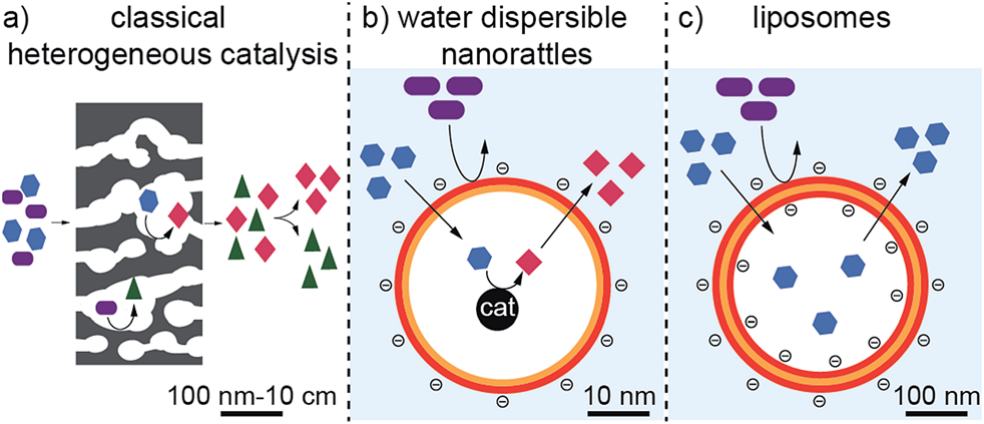
Schematic representation of classical heterogeneous catalysis (a), liposomes (c) and a vehicle combining properties of these two systems, namely good dispersibility and mobility in water and selective catalytic activity (b).

Traditional catalysts, such as porous carbon^1^ and zeolites^2^ are well known for their ability to perform size- and shape-selective catalysis.^3^ These catalysts possess catalytic active species in their microporous structures. The small pore sizes of the catalysts are responsible for their size- and shape-selectivity.^3^ This is in contrast to classical catalysis, where the catalytic conversion of several different sized or shaped reagents often leads to a mixture of products (Fig. 1a). However, these materials are usually rather large in size and therefore possess low mobility. Higher mobility of heterogeneous catalysts can be achieved by lowering their size to the nanometer scale. For instance, metallic nanoparticles are very well known to efficiently catalyse chemical reactions. Enclosing such nanoparticles in porous shells can give them size- and shape-selective properties as the capsule can sieve and select incoming or leaving molecules, comparable to zeolites and porous carbons.^4^–^6^ In addition, the compartmentation allows locating the chemical reaction to a specific space in which appropriate conditions could be created (concentrations, functional groups, *etc.*). Such nanostructures are called nanorattles.^7^–^10^ They consist of one or several particles enclosed by a shell. Their core is usually made of metals^4^,^5^,^11^–^17^ and oxides.^18^,^19^ Frequently used materials for the shell are silica^6^,^18^,^20^–^22^ and carbon^16^,^17^,^23^–^25^ but also polymers^13^,^14^,^26^,^27^ or metals.^28^,^29^ The drawback of nanorattles is that they are usually not dispersible in aqueous solutions; especially carbon nanorattles often possess a hydrophobic surface because of the high temperatures required in the production process for carbonization.^30^ This restricts the use of most carbon nanorattles to non-polar solvents and applications in aqueous systems are rare.

On the other side of the spectrum, the most prominent examples of water compatible capsules are certainly liposomes.^31^,^32^ These self-assembled bilayer structures can incorporate and release a cargo, can be surface functionalized to tune their properties and are highly dispersible and mobile in aqueous solutions (Fig. 1c).^31^,^32^ They find many applications for the administration of nutrients and pharmaceutical drugs and can even be used to selectively accommodate a specific substance and therefore can find application in detoxification.^33^ However, liposomes are not used for catalytic transformations. Although it has been shown that metallic nanoparticles can be incorporated in liposomes,^34^–^38^ these nanoparticles are not used for catalysis but for triggered release,^34^ fluorescence properties^35^ or as contrast agents.^39^ Furthermore, the rather low physical and chemical stability of liposomes brings some limitations, especially for applications in more demanding environments.

Therefore, a shell, which is more stable than liposomes but still possesses their favorable properties, namely the small size, the good dispersibility in aqueous systems and the sieving properties of the membrane to select the substances that can enter and leave the cavity, would be highly desirable. If catalytic active species could be incorporated to such a hollow sphere, it would be possible to achieve a very well water-dispersible catalyst, which could perform selective catalysis arising from the restricted accessibility (Fig. 1b).

**Fig. 2:**
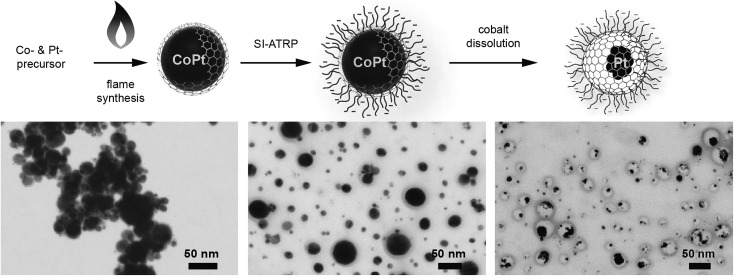
Synthesis of hollow carbon nanocapsules with enclosed platinum: carbon-coated cobalt–platinum-alloy nanoparticles are synthesized by reducing flame spray synthesis. After surface functionalization *via* surface-initiated atom transfer radical polymerization (SI-ATRP) to achieve favourable surface properties, the cobalt is removed by selective dealloying in order to obtain platinum nanorattles.

Here, we show the synthesis of nanoparticles with a bimetallic alloy core and the subsequent selective dissolution (dealloying) of the less noble metal. This reveals a rattle-type particle, consisting of a hollow porous carbon capsule, which encloses the remaining noble metal particles (Fig. 2). This capsule is well dispersible in aqueous systems due to the selective hydrophilic functionalization of the outer capsule wall and performs size-selective catalysis.

## Results and discussion

### Synthesis and characterization

The synthesis starts with carbon-coated cobalt–platinum-alloy nanoparticles (C/CoPt), which were produced by reducing flame spray synthesis. Reducing flame spray synthesis is known for the ability to produce numerous core/shell particles with cores consisting of various metals or even multicomponent alloys.^40^ The platinum and cobalt precursors were mixed in a ratio resulting in the desired Co–Pt-alloy nanoparticles. Addition of acetylene during the formation process allowed the *in situ* formation of three to four graphene-like sp^2^-hybridized carbon layers surrounding the metallic core as previously shown by high resolution TEM, ^13^C-MAS-NMR and Raman spectroscopy.^41^,^42^ Particles with different platinum contents were synthesized (desired loadings: 1 wt%, 10 wt% and 20 wt%). The successful production of such C/CoPt particles with different platinum loadings was confirmed by X-ray diffraction (XRD) showing a peak shift for the C/CoPt particles with increasing platinum content (Fig. S1†). Thermogravimetric analysis and inductively coupled plasma time-of-flight mass spectrometry (ICP-TOFMS) indicated that about 50–70% of the expected platinum amount is present in the samples (Fig. S2, Tables S2–S4†). Diameter analysis of scanning electron microscopy (SEM) images of the various particles revealed a similar average particle diameter of around 20 nm independent of Pt loading (Fig. S3 for STEM/SEM images and Fig. S4 for particle size distributions†). Cobalt was chosen as the second metal of the alloy because of its good alloying behaviour with platinum^43^,^44^ and the introduction of magnetic properties.^45^ The latter facilitate the surface functionalization by magnetic sample purification in later steps.

**Scheme 1:**
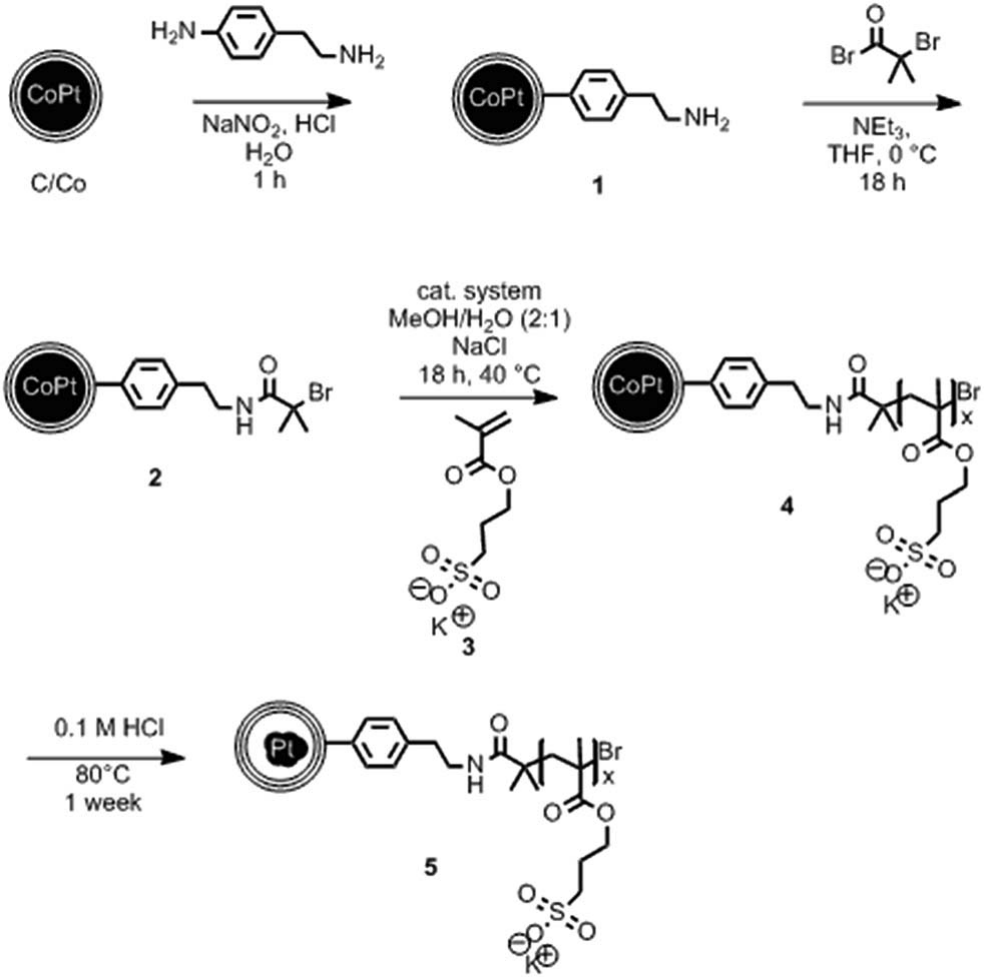
Covalent surface functionalization of carbon-coated cobalt–platinum-alloy nanoparticles (C/CoPt) and subsequent formation of carbon nanorattles containing platinum in the interior (**5**). Diazonium chemistry (**1**) and amidation were used to generate an initiator (**2**) for surface-initiated atom transfer radical polymerization (SI-ATRP) of 3-sulfopropyl methacrylate potassium salt (SPM, **3**) to yield C/CoPt@pSPM (**4**). Subsequent selective dissolution of the cobalt from the core under acidic conditions and elevated temperature yields carbon nanorattles containing platinum (**5**). Cat. system: copper(ii)bromide (CuBr_2_), 2,2′-bipyridine, l-ascorbic acid.

In order to achieve well dispersible and individually appearing nanorattles in aqueous systems, the hydrophilic 3-sulfopropyl methacrylate polymer was covalently bound to the carbon layer *via* atom transfer radical polymerization (Scheme 1 and Table S1†). The polymer introduces steric and electrostatic repulsion between the individual particles such that agglomeration due to hydrophobic interaction between the graphene-like carbon surfaces is prevented.^46^ Forbearance of this surface functionalization before acidic dealloying would result in an inseparable mixture of nanorattles and still intact nanoparticles and thus, hinder the isolation and analysis of the nanorattles (Fig. S5†).

The selective dissolution of the cobalt by hydrochloric acid at elevated temperatures for one week allowed the production of rattle-type hollow carbon nanospheres containing platinum in their interior (Fig. 2). This selective etching of the less-noble metal from an alloy, leaving behind a nanoporous framework of the remaining metal, is called selective dealloying.^47^–^49^ Generally, the resulting nanoporous metal foams of such dealloying processes can be used in different applications, one of them is catalysis.^49^ RANEY® nickel,^50^ for example, is used as reagent and catalyst in industrial processes and organic synthesis.^51^

**Fig. 3:**
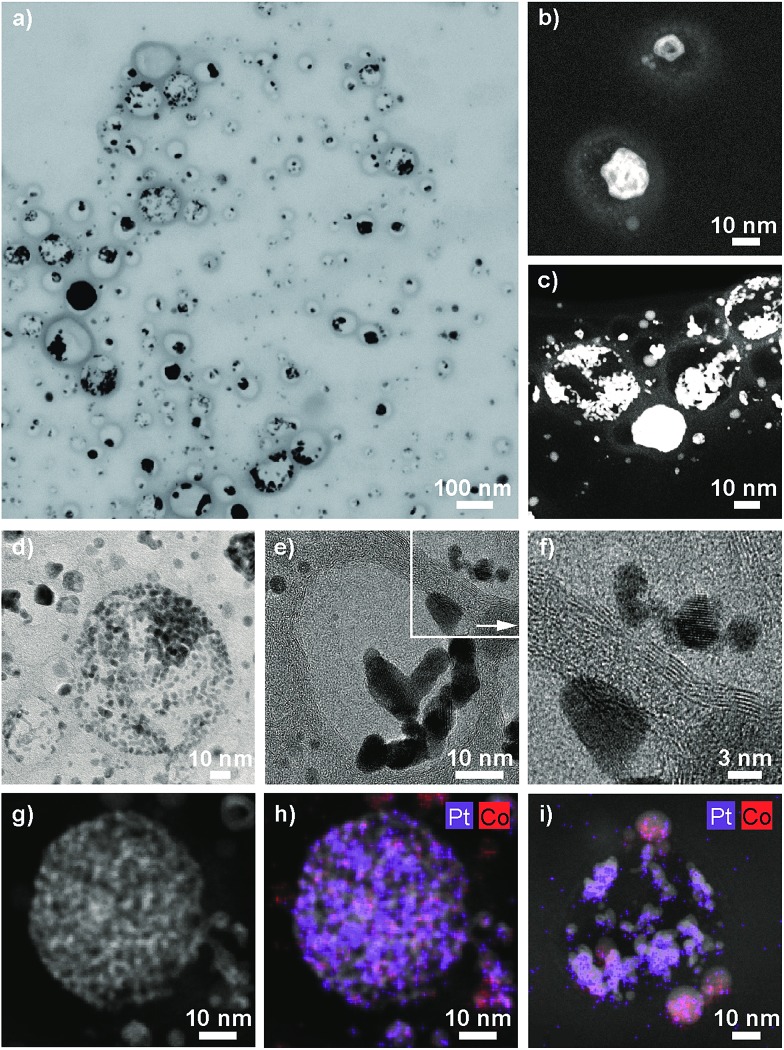
Scanning transmission electron micrograph (STEM, a), transmission electron micrographs (TEM, b–g) and energy-dispersive X-ray spectroscopy maps (EDX, h, i) of platinum nanorattles derived from carbon-coated cobalt–platinum-alloy nanoparticles (C/CoPt) containing 20 wt% platinum.

**Fig. 4:**
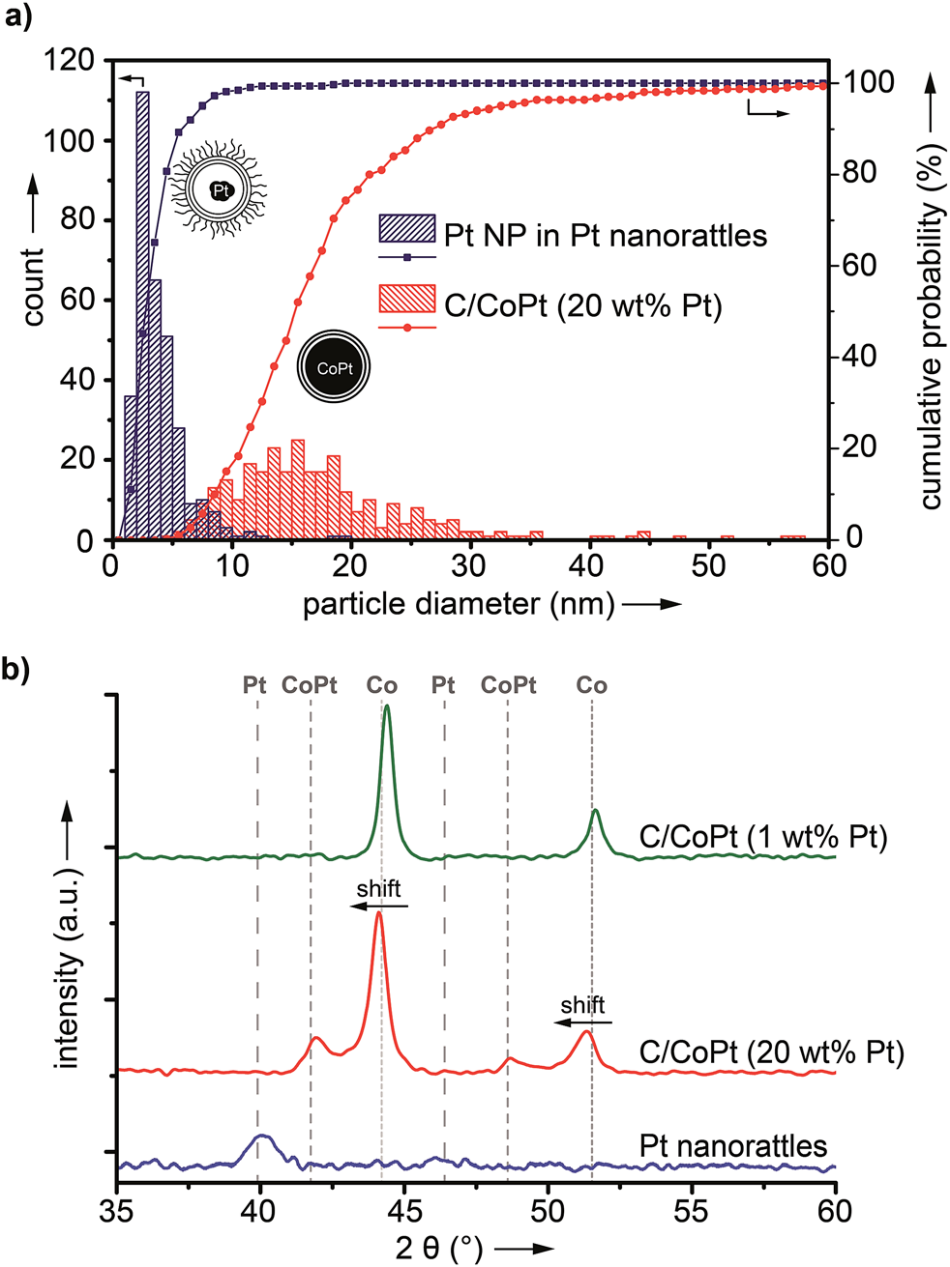
Number-weighted size distribution of platinum nanoparticles in platinum nanorattles and carbon-coated cobalt–platinum-alloy nanoparticles (C/CoPt) containing 20 wt% platinum, obtained from microscopy images (a) and X-ray diffraction patterns of C/CoPt containing 1 and 20 wt% platinum and of platinum nanorattles derived from C/CoPt (20 wt% Pt) (b). The intensity of the Pt nanorattles pattern is fivefold increased to improve visibility.

ICP-OES measurements of the leaching solutions of the selective dissolution of the cobalt from the particles revealed a leaching yield of about 30% (Tables S5 and S6†). Electron microscopy images clearly showed the success of the partial dissolution of the metal core (Fig. 3, S6 and S7†). X-ray spectroscopy (EDX) mapping revealed that the vast majority of the metal left in the capsules was platinum (Fig. 3h, i and S8†). Cobalt was still detectable in the sample but this seemed to originate from non-leached particles. Stereoscopic images of the nanorattles (Fig. S9†) suggest that the platinum nanoparticles stick to the inner surface of the carbon shell. Leaching of the cobalt from the core is possible because of pore formation in the carbon layers during the acidic treatment.^52^ The acidic treatment seems to not destroy the integrity of the carbon laysers^41^,^42^ enclosing the platinum particles as can be seen in Fig. 3e and in higher magnification in Fig. 3f, where the individual graphene-like layers are visible. STEM images of the different rattles produced from the particles having different platinum loadings (1 wt%, 10 wt% and 20 wt%) are shown in Fig. S10.† All further analysis was performed with nanorattles derived from C/CoPt (20 wt% Pt) nanoparticles. The success of the dealloying step was further confirmed by comparing the size distribution of the C/CoPt (20 wt% Pt) nanoparticles before the dissolution process and the size distribution of the remaining platinum nanoparticles within the hollow shells of the nanorattles after cobalt removal (Fig. 4a). The mean number-weighted particle diameter decreased from 18 ± 10 nm for C/CoPt to a mean platinum particle diameter of 3.8 ± 2.2 nm within the nanorattles. XRD of the nanorattles showed the complete disappearance of cobalt and the appearance of a platinum peak after the dealloying step (Fig. 4b). This measurement of the bulk substance reinforced what had been observed in EDX maps, namely that after selective dealloying the majority of the remaining metal was platinum. Using the Scherrer formula,^53^ the crystal size of the platinum can be determined from the XRD peak-width, resulting in a crystal size of 7.7 nm. Comparing this crystal size to the volume-weighted mean diameter determined by microscopy images (5.3 nm) leads to the assumption that the platinum nanoparticles are single crystals (Table S7†).

Thermogravimetric analysis of the nanorattles derived from C/CoPt (20 wt% Pt) showed a relative mass loss of 67 wt% after heating to 600 °C, indicating that 33 wt% of the sample is metallic (Fig. S11†). This finding is in agreement with ICP-TOFMS measurements of the nanorattles, which revealed a platinum and cobalt content of 18.2 wt% and 6.3 wt%, respectively (total metal content: 25.5 wt%; Table S8†).

### Size-selective catalysis

**Fig. 5:**
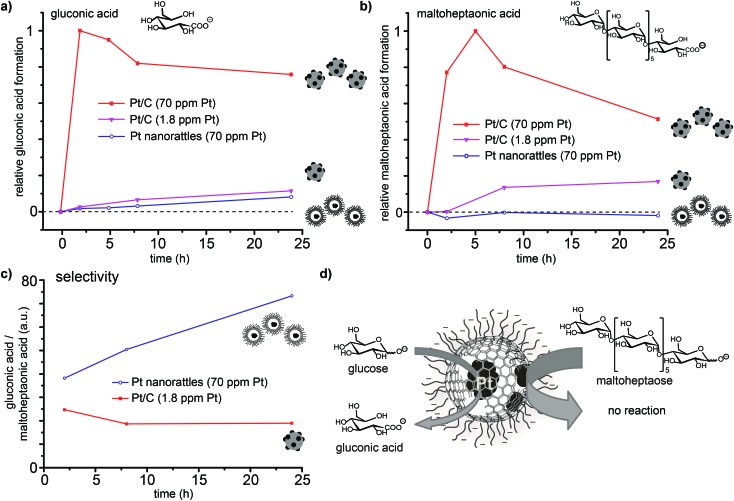
Platinum catalysed oxidation of glucose to gluconic acid (a) and maltoheptaose to maltoheptaonic acid (b) in one pot using commercially available platinum on carbon (Pt/C) in two different concentrations and platinum nanorattles. Depicting the results as ratio of the relative formation of gluconic acid and maltoheptaonic acid (non-normalized LC/MS peak) (c) exemplifies the selectivity of the nanorattles in favour of the gluconic acid formation and with it the size selectivity of the nanorattles catalyst (d).

In order to confirm that platinum is solely present in the spheres' interior, the platinum catalysed oxidation of two differently sized reducing sugars (glucose and maltoheptaose) was performed. The pores created in the carbon shell during acidic dissolution,^52^ should only allow small molecules to enter the spheres and therefore be oxidized, while the larger molecules would be kept outside and remain unaffected. The oxidation of reducing sugars on platinum provides a good model reaction, as the substrate size can be changed easily by using oligomers of the corresponding sugar (in this case the heptamer of glucose; Scheme S2†). The performed surface functionalization of the outer capsule wall accounts for the good dispersions of the nanorattles in the aqueous reaction media. The reaction was performed by adding the catalyst to an alkaline solution of glucose and maltoheptaose in one pot at room temperature. Commercially available platinum on carbon (Pt/C) was used to perform the same reaction as a control. Mass spectrometry measurements of the reaction mixture over time revealed that while the oxidation of both glucose and maltoheptaose occurred in the presence of Pt/C, only the oxidation of glucose took place in the presence of the nanorattles (Fig. 5). STEM and SEM images of the Pt nanorattles after catalysis showed no change in structure (Fig. S12†). However, the relative conversion of glucose to gluconic acid in the presence of the nanorattles is much lower compared to the commercially available Pt/C catalyst. The drastic decline in the reaction rate may be an indication of diffusion limitation. This is not very surprising considering that a substrate molecule first has to diffuse through the polymer layer and the carbon capsules pore before it reaches the platinum catalyst and gets oxidized. Another reason for the low reaction rate could be the broad size-distribution of the platinum nanoparticles.^54^,^55^ Control reactions with empty nanorattles^56^ (no platinum inside the nanoshells) and spherical carbon nanoparticles showed no oxidation of glucose and maltoheptaose (Fig. S13†). A control reaction with platinum nanoparticles (which were prepared by reducing H_2_PtCl_6_ with NaBH_4_) in the presence of empty nanorattles (Fig. S14†) indeed showed catalytic activity but no size selectivity (Fig. S13†).

In order to ensure that the absence of maltoheptaose oxidation in the presence of platinum nanorattles does not arise from a kinetic effect, the reaction was also repeated with a lower concentration of Pt/C in order to reach a similar relative conversion as with the nanorattles. This experiment showed that with lower concentrated Pt/C the oxidation of maltoheptaose took place, whereas with the nanorattles still no oxidation of maltoheptaose was detectable (Fig. 5). While the commercial Pt/C catalyst shows no size-selectivity, nanorattles show a clear selectivity in favour of glucose as depicted in Fig. 5c. The absence of catalytic activity for the oxidation of maltoheptaose reinforces the predominance of encapsulated platinum particles as active sites. It furthermore shows that the nanorattles can be regarded as a size-selective catalyst, where the diffusion of the molecules through the polymer and the porous carbon shell adds the size selectivity to the platinum catalyst.

## Conclusions

In summary, selective dealloying of cobalt–platinum-alloy nanoparticles, coated with graphene-like carbon, led to rattle-type nanostructures consisting of outer-surface functionalized carbon nanoshells with incorporated platinum nanoparticles. These particles combine the advantageous properties of liposomes, with their good dispersion stability and high mobility in aqueous solutions, and carbon spheres (*e.g.* fullerenes) with their chemical integer structure and their high stability. Platinum catalysed oxidation of differently sized sugars showed that the nanorattles can conduct size-selective catalysis. Such size- and shape-selectivity is well known from other catalytic systems, such as zeolites^57^,^58^ and metal organic frameworks (MOFs).^59^ The capability to perform size-selective catalysis is in this case reduced to the single particle level. The outer surface functionalization and the small size of these nanorattles lead to the good dispersibility in water and the high mobility of this catalyst.

It can be envisioned that the here presented concept of size-selective catalysis with carbon nanorattles synthesized *via* dealloying carbon-coated metal-alloy nanoparticles could be extended regarding the enclosed metal, the capsule and the shell porosity. It is therefore imaginable that the route described above provides access to a wide range of different nanostructured materials with great potential for a variety of applications even visionary ones, such as target specific *in vivo* catalysis.^60^

## Conflicts of interest

There are no conflicts to declare.

## Supplementary Material

Supplementary information
