# Supplementary material for: Water dispersible surface-functionalized platinum/carbon nanorattles for size-selective catalysis
Source: Chem Sci. 2017 Oct 30;9(2):362–7. doi: 10.1039/c7sc03785f (PMC5868313; doi:10.1039/c7sc03785f)
Supplement: Supplementary file 1 [file SC-009-C7SC03785F-s001.pdf]

Electronic Supplementary Material (ESI) for Chemical Science.
This journal is © The Royal Society of Chemistry 2017

Supporting Information

Water dispersible surface-functionalized platinum/carbon nanorattles for size-selective catalysis

*Corinne J. Hofer, Robert N. Grass, Elia M. Schneider, Lyndsey Hendriks, Antoine F. Herzog, Martin Zeltner, Detelef Günther, and Wendelin J. Stark**

Table of Contents

1 Materials and General Methods 3
2 Synthesis of platinum nanorattles 4
2.1 Elemental microanalysis results 5
3 Characterization of carbon-coated cobalt-platinum-alloy nanoparticles (C/CoPt) 6
3.1 X-ray diffraction pattern (XRD) 6
3.2 Thermogravimetric analysis 7
3.3 Inductively coupled plasma time-of-flight mass spectrometry (ICP-TOFMS) 8
3.4 Scanning (transmission) electron micrographs (STEM/SEM) 8
3.5 Particle size distribution and cumulative probability 9
4 Acidic dissolution 10
5 Characterization of platinum nanorattles 11
5.1 Scanning (transmission) electron micrographs (STEM/SEM) 11
5.2 Transmission electron micrographs (TEM) 12
5.3 Energy-dispersive X-ray spectroscopy (EDX) 13
5.4 Stereoscopic images 13
5.5 Scanning (transmission) electron micrographs (STEM/SEM) 14
5.6 X-ray diffraction pattern (XRD) 15
5.7 Thermogravimetric analysis 15
5.8 Inductively coupled plasma time-of-flight mass spectrometry (ICP-TOFMS) 16
6 Size-selective catalysis 16
6.1 Catalytic oxidation of glucose and maltoheptaose by platinum catalysts 16
6.2 Scanning transmission electron micrographs (STEM) of Pt nanorattles after usage as catalyst 17
6.3 Results of control reactions 18
6.4 Formation of platinum nanoparticles in the presence of nanobubbles (Pt NP & HCNB) 19
7 References 19

1 Materials and General Methods

Dispersions of nanoparticles were produced with the aid of an ultrasonic bath (Bandelin Sonorex) and a high sheer mixer (T10 basic ULTRA-TURRAX (IKA)). For centrifugation a 3-30KS centrifuge (Sigma) was used. The carbon nanorattles were filtered through Amicon Ultra-15 or -4 Centrifugal Filter Units (50 kDa, Merck). The nanoparticles were analyzed by elemental microanalysis ELEMENTAR (Elementar Analysensysteme). X-ray diffraction (XRD) patterns were recorded on an X'Pert PRO-MPD diffractometer (Cu-Kα radiation, X'Celerator linear detector system; PANalytical, Netherlands) and inductively coupled plasma atomic emission spectroscopy (ICP-OES) by ULTIMA ICPOES. Transmission electron microscopy (TEM) was measured with a F30 (Tecnai F30 ST (FEI, FEG, 300 kV)), scanning transmission electron microscope (STEM) on a NovaNanoSEM 450 (FEI, operated at 30 kV) with a HAADF detector. EDX was performed on a HD2700CS. For both, the particles were deposited on a carbon/copper grid.

The analyte concentration (platinum and cobalt in nanorattles) was determined using an inductively coupled plasma time-of-flight mass spectrometer (icpTOF, TOFWERK AG). No sample treatment was conducted and samples were diluted in ultra-high purity (UHP) water (Millipore, Billerica, Massachusetts, USA). Platinum, cobalt and iridium stock solutions were prepared from single-element standard solutions (Inorganic Ventures, Christiansburg, Virginia, USA). Subsequent dilutions were prepared with a solution of 2% HNO_3 and 2% HCl (Traceselect Ultra, Fluka) in UHP water. All dilutions were carried out gravimetrically using an electronic balance (Mettler AE240, Mettler-Toledo, Grefensee, Switzerland). Final solutions of platinum and cobalt with concentrations ranging from 1 ng/g to 500 ng/g were used for external calibrations. 10 ng/g Iridium was spiked in the calibrations solutions as well as in the diluted samples as internal standard to monitor instrumental drift and analyte response. 10000 TOF extractions were integrated for one data point and 200 data points were collected for a minute. Hence, 200 data points each integrated over 300 ms were used to determine the average and standard deviation.

Mass spectrometry of the product of the platinum catalyzed oxidation of glucose and maltoheptaose was performed using an Agilent 1260 Infinity Quadrupole 6130 LC/MS. The parameters for the direct injection method (also mass spectrometer parameters) are as follows: 5 µl injection, 0.1 mL/min flow, a solvent mix of 50/50 (A: NH_4Ac 13 mM at pH7, B: MeCN), fragmentor = 200, capillary voltage 4000 V +/-, drying gas flow = 12 l/min, nebulizer pressure = 35 psig, drying gas temperature = 250 °C. Simultaneous electrospray ionization (ESI) in positive and negative mode was performed with a fast switching dielectric capillary which allowed for injection of the sample only once. Single ion monitoring (SIM) in the negative mode was done for relative quantification of the products of the catalysis, namely gluconic acid (195 m/z) and maltoheptanonic acid (583 m/z).

The particle size distribution was characterized by optically measuring the diameter of at least 200 particles per sample in STEM/SEM images.

Chemicals: Platinum acetylacetonate (≥ 98%, ABCR-Chemicals), 2-ethylhexanoic acid (≥ 99%, Sigma-Aldrich), tetrahydrofuran (THF) (≥ 99%, Fisher Scientific), hydrochloric acid (HCl, Sigma-Aldrich, 37%), 3-(2-Methylprop-2-enoyloxy)propane-1-sulfonic acid potassium salt (SPM, ABCR), copper(II)bromide ($CuBr_2$, Fluka, 99%), 2,2′-bipyridine (bipy, ABCR,

99%), L-ascorbic acid (Sigma-Aldrich, 99%), sodium chloride (NaCl, Merck, for analysis), D-Glucose (anhydrous for analysis, Axon Lab AG), Maltoheptaose (95%, ABCR), platinum on carbon (Pt/C, 5 wt%, matrix activated carbon support, Sigma-Aldrich), potassium hydroxide (KOH, pellets, 85%, Sigma-Aldrich) hexachloroplatinic (IV) acid hydrate (H_2PtCl_6, Aldrich Fine Chemicals, 38% Pt basis) and sodium tetrahydroborate ($NaBH_4$, Fluka, 97%) were used as obtained if not stated otherwise.

2 Synthesis of platinum nanorattles

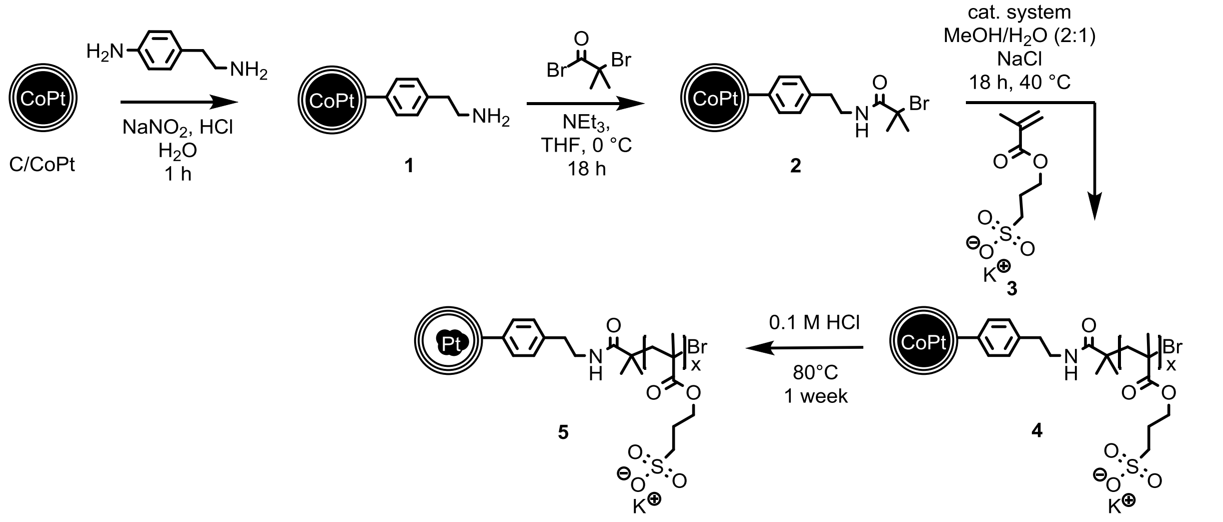

Scheme S1 Covalent surface functionalization of carbon-coated cobalt-platinum-alloy nanoparticles (C/CoPt) and subsequent formation of carbon nanorattles containing platinum in the interior (**5**). Diazonium chemistry (**1**) and amidation were used to generate an initiator (**2**) for surface-initiated atom transfer radical polymerization (SI-ATRP) of 3-sulfopropyl methacrylate potassium salt (SPM, **3**) to yield C/CoPt@pSPM (**4**). Subsequent selective dissolution of the cobalt from the core under acidic conditions and elevated temperature yields carbon nanorattles containing platinum (**5**). Cat. system: copper (II)bromide ($CuBr_2$), 2,2'-bipyridine, L-ascorbic acid.

Production of C/CoPt-nanoparticles. Platinum acetylacetonate (0.435 g) was dissolved in a mixture of 2-ethylhexanoic acid and tetrahydrofuran (60 mL, wt/wt 1:1) to give a brownish precursor solution. The metal content of the platinum precursor was determined by adding small amounts of precursor into Erlenmeyer-flasks and burning of the organic contents by heating to 450 °C for 4 h. By measuring the mass of the residues and knowing the initial amounts of precursor which was burnt, the metal content was calculated (1.37 wt% Pt). The cobalt precursor (cobalt 2-ethylhexanoate, 65% in mineral spirits, 12% Co, ABCR) was diluted by THF (precursor / THF 1:2). The platinum and the cobalt precursors were mixed at a ratio to target 20 wt%, 10wt% and 1 wt% platinum in cobalt. The carbon-coated cobalt platinum nanoparticles were produced in a one step process by reducing flame spray pyrolysis under an oxygen-free atmosphere as described by Grass et al.[1]

Synthesis of C/CoPt@initiator (2) was performed as described elsewhere.[2]

Synthesis of C/CoPt@pSPM (4). The procedure was adapted from literature.[3] All reaction steps were performed under a protective nitrogen atmosphere. The monomer solution was prepared by dissolving 3-(2-Methylprop-2-enoyloxy)propane-1-sulfonic acid potassium salt (SPM, **3**) (8.6 g, 34.9 mmol) in MeOH/H_2O (2:1, 12 mL) and consecutive degassing by nitrogen bubbling for 30 minutes. $CuBr_2$ (10 mg, 0.045 mmol), 2,2'-bipyridine (54 mg, 0.35

mmol), L-ascorbic acid (60 mg, 0.34 mmol) and NaCl (90 mg, 1.54 mmol) were added to the solution and it was degassed for further 5 minutes. C/Co@initiator (**2**) (500 mg) were placed in a Schlenk flask and degassed (3 × high vacuum pump / N_2 refill cycles). The monomer solution was then added by syringe. The reaction mixture was exposed to sonication for 10 minutes to obtain a homogeneous dispersion. It was then stirred for 18 hours at 40 °C. The poly-SPM functionalized nanoparticles (C/CoPt@pSPM, **4**) were magnetically separated. After magnetic decantation, the particles were washed three times with water. Acetone (twice the volume of the washing water) was used to destabilize the particles. It was further washed with ethanol, ethyl acetate and acetone, twice each. After each washing procedure (sonication for 3 minutes in solvent) the particles were recovered by the external magnet and the washing solvent was drained. The nanoparticles were dried in a vacuum oven at 50 °C.

Synthesis of polymer coated carbon nanorattles containing Pt (5). Pestled C/CoPt@pSPM (**4**) (50 mg) were added to hydrochloric acid (0.1 M, 50 mL) and dispersed by ultrasonication for 15 minutes and subsequent stirring by the aid of a high sheer mixer. The dispersion was then magnetically stirred at 1000 rpm and heated to 80 °C for one week. The nanorattles and non-dissolved cobalt-platinum particles were separated from each other by magnetic separation of the latter and subsequent draining of the supernatant containing the nanorattles. The nanorattles were then collected from the reaction solvent by the aid of amicon ultra-15 centrifugal filter units and then washed five times by adding water to the concentrate, shaking and subsequent filtration.

2.1 Elemental microanalysis results

Table S1 Elemental microanalysis results for carbon, nitrogen, sulfur and bromine content of C/CoPt (20 wt% Pt), **1**, **2** and **4**.

compound	C (wt%)	N (wt%)	S (wt%)	Br (wt%)	loading (mmol/g)	units / starter
C/CoPt (20 wt% Pt)	3.91	0.03	0	n.a.	-	
**1**	5.54	0.25	0	n.a	0.17[a] / 0.18[b]	
**2**	5.94	0.26	n.a.	0.86	0.08[a] / 0.11[c]	
**4**	16.27	0.36	3.98	n.a	1.23[a] / 1.24[d]	15[a] / 11[c]

[a]: calculated with respect to increase in carbon content, [b]: calculated with respect to increase in nitrogen content, [c]: calculated with respect to increase in bromine content, [d]: calculated with respect to increase in sulfur content, n.a.: not analyzed.

3 Characterization of carbon-coated cobalt-platinum-alloy nanoparticles (C/CoPt)

3.1 X-ray diffraction pattern (XRD)

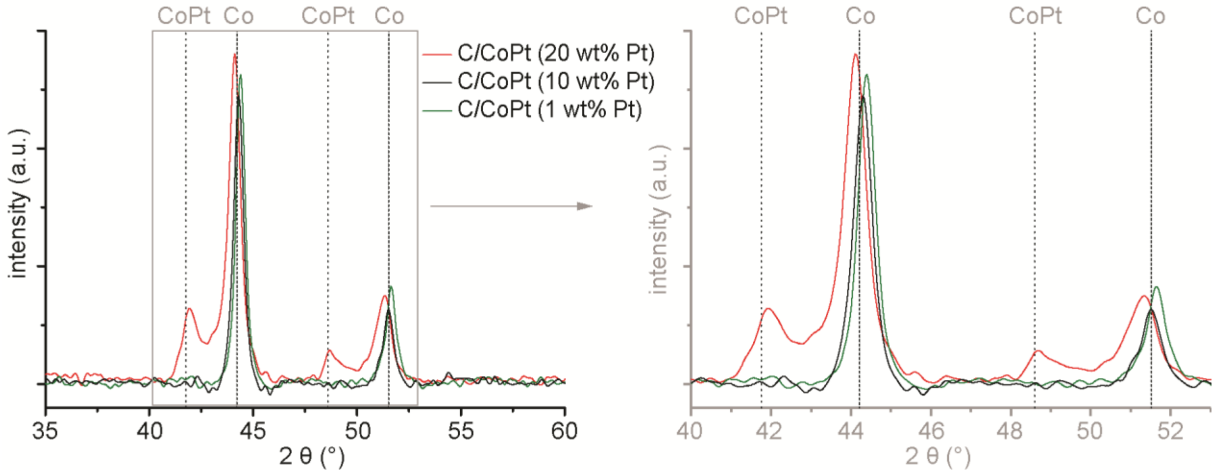

Fig. S1 X-ray diffraction pattern of carbon-coated cobalt-platinum-alloy nanoparticles (C/CoPt) containing 1 wt%, 10 wt% and 20 wt% platinum. Reference lines drawn in grey are taken from pdf_00-015-0806 for Co and pdf_03-065-8970 for CoPt.

Table S2 Measured platinum content of carbon-coated cobalt-platinum-alloy nanoparticles (C/CoPt) with different platinum contents measured by quantitative analysis of X-ray diffraction patterns.

sample	platinum content
C/CoPt (20 wt% Pt)	10 wt%
C/CoPt (10 wt% Pt)	5 wt%
C/CoPt (1 wt% Pt)	n.d.

n.d: not detectable

3.2 Thermogravimetric analysis

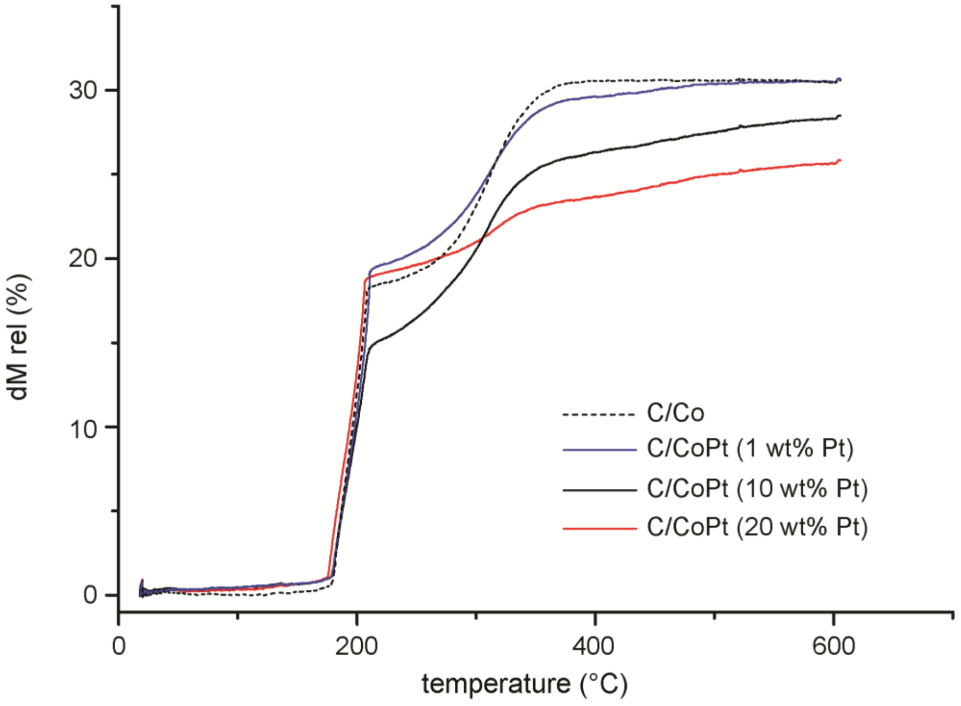

Fig. S2 Relative mass gain of carbon-coated cobalt nanoparticles (C/Co) and carbon-coated cobalt-platinum-alloy nanoparticles (C/CoPt) with different platinum contents, measured by thermogravimetry in air. C/Co nanoparticles showed a mass gain of 30.6%, C/CoPt (1 wt% Pt) 30.6%, C/CoPt (10 wt% Pt) 28.5% and C/CoPt (20 wt% Pt) 25.8% upon heating to 600 °C.

From the mass gain in thermogravimetric analysis the amount of platinum in the C/CoPt particles can be estimated. By knowing the weight percentage of carbon within the particles from elemental analysis and the mass gain upon oxidation of the cobalt, it is possible to calculate the amount of platinum present in the sample via the following equation:

$$(1-C)\cdot(Pt+(1-Pt))\cdot 1.36 = 1+\Delta\mathrm{m}_{\mathrm{ox}}$$

$$Pt = \frac{2.78\cdot(1+\Delta\mathrm{m}_{ox})}{C-1} + 3.78 \qquad \text{(eq. S1)}$$

where C is the weight part of the particles being carbon, Pt is the weight part of the particles being platinum and the factor 1.36 resulting from the mass gain of cobalt upon oxidation (Co → Co_3O_4).

Calculating the platinum amount of the particles via equation S1 leads to the results shown in Table S3.

Table S3 Calculated amount of platinum in different carbon-coated cobalt-platinum-alloy nanoparticles (C/CoPt) derived from results of thermogravimetric analysis via equation S1.

particles	calculated platinum content
C/CoPt (20 wt% Pt)	14 wt%
C/CoPt (10 wt% Pt)	4 wt%
C/CoPt (1 wt% Pt)	-5 wt%

3.3 Inductively coupled plasma time-of-flight mass spectrometry (ICP-TOFMS)

Table S4 Estimated and measured platinum and cobalt content of C/CoPt@pSPM (**4**) nanoparticles measured by inductively coupled plasma time-of-flight mass spectrometry (ICP-TOFMS).

species	estimated metal content [a)]	measured metal content
C/CoPt@pSPM (**4**) (20 wt% Pt)	Pt : 12.7%	Pt: 8.7%
	Co : 50.7%	Co: 25.3%

[a)]: calculated from the wt% of the organic part of the C/CoPt@pSPM (**4**) particles, determined by elemental microanalysis, with the assumption of total cobalt dissolution and that the platinum content is the one which was aimed in the particle production process (meaning 20 wt% Pt).

3.4 Scanning (transmission) electron micrographs (STEM/SEM)

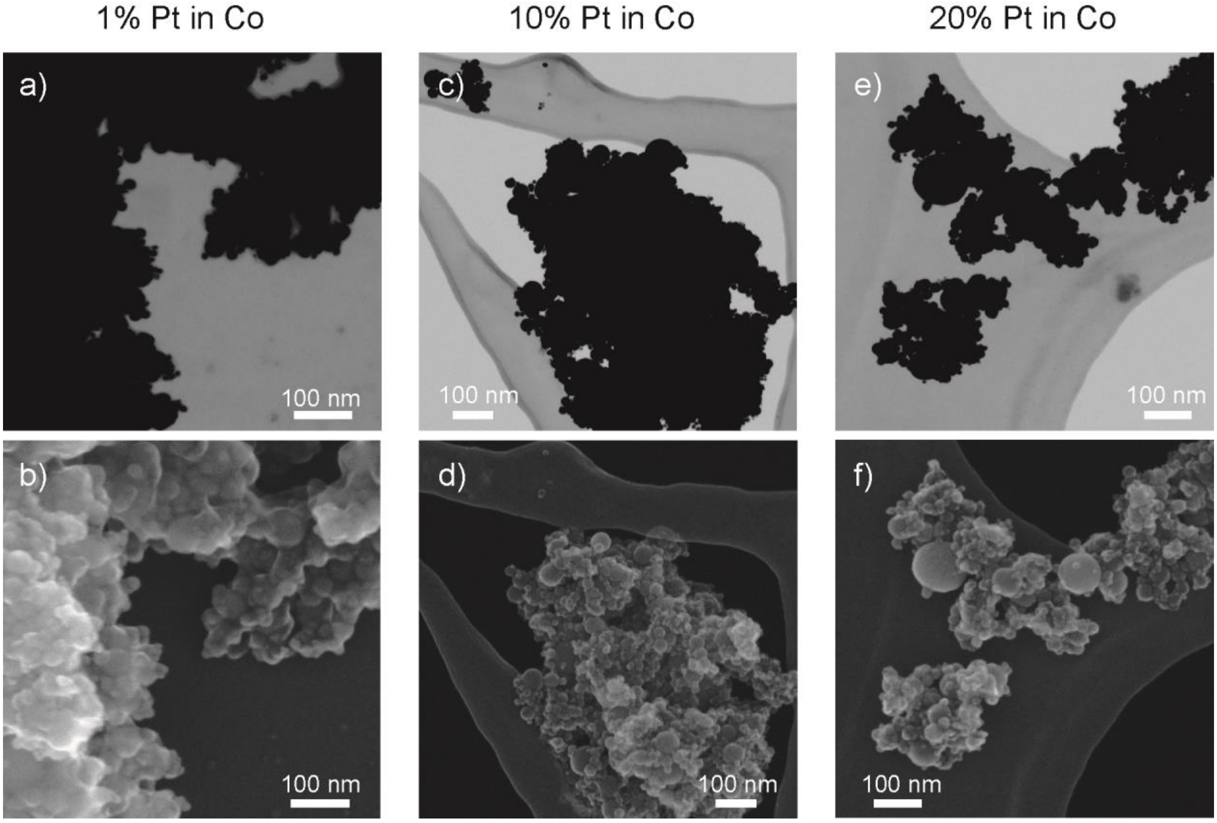

Fig. S3 Scanning transmission electron microscopy images (STEM, a, c and e) and scanning electron microscopy images (SEM, b, d and f) of carbon-coated cobalt-platinum nanoparticles (C/CoPt) containing different amounts of platinum 1 wt%, 10 wt%, 20 wt%).

3.5 Particle size distribution and cumulative probability

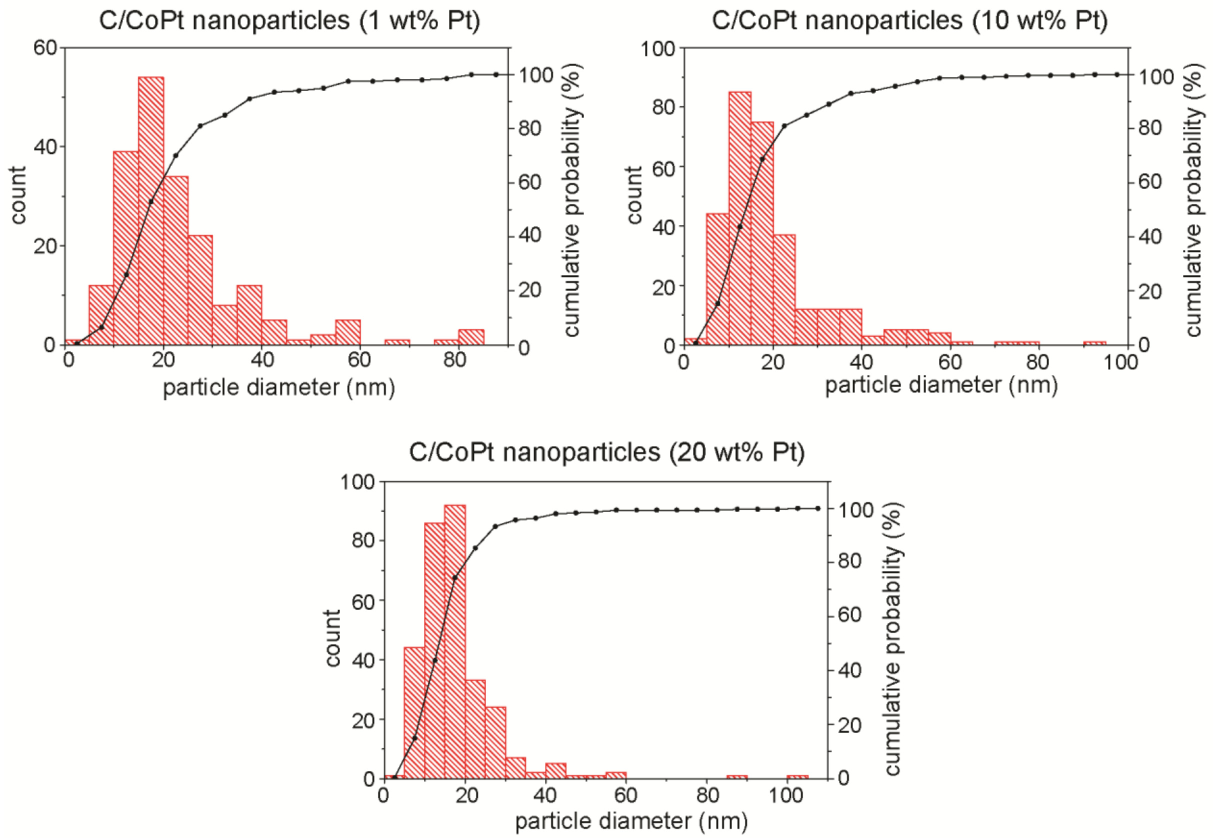

Fig. S4 Particle size distribution and cumulative probability of carbon-coated cobalt-platinum-alloy nanoparticles (C/CoPt) containing 1 wt%, 10 wt% and 20 wt% platinum measured by diameter analysis in STEM pictures. An average diameter of 23 ± 14 nm for C/CoPt (1 wt% Pt), 19 ± 13 nm for C/CoPt (10 wt% Pt) and 18 ± 10 nm for C/CoPt (20 wt% Pt) was found by measuring 200, 300 and 300 particles, respectively.

4 Acidic dissolution

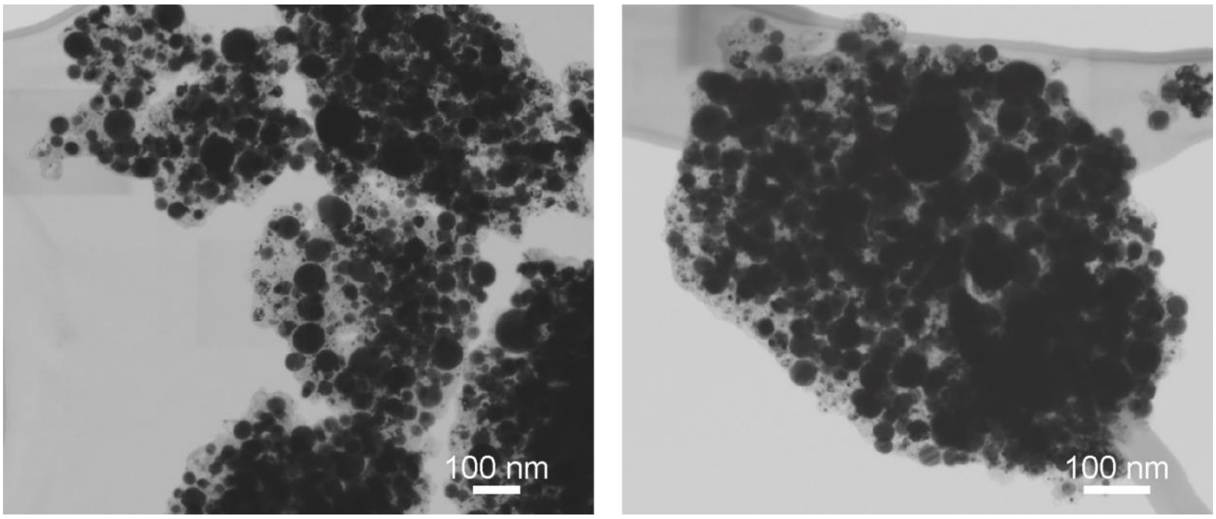

Fig. S5 Scanning transmission electron microscopy (STEM) of a mixture of leached (nanorattles) and non-leached carbon-coated cobalt-platinum-alloy nanoparticles (C/CoPt (20 wt% Pt)) without surface functionalization.

Table S5 Calculated and found concentration of cobalt and platinum ions by ICP-OES in the supernatant of the reaction mixture after magnetic separation of the non-dissolved cobalt-platinum-alloy nanoparticles and filtration of the nanorattles.

sample	$[Co^{2+}]$ (ppm)		[Pt-ion] (ppm)	
	calculated[a]	found[b]	calculated[a]	found[b]
**5** (1 wt% Pt)	847	254	8.6	0.3
**5** (20 wt% Pt)	444	143	111.0	0.3

[a]: calculated from the wt% of cobalt and platinum in the sample (determined by elemental microanalysis) and the amount of non-dissolved, magnetically removed cobalt-platinum particles under the assumption that full cobalt dissolution occurred, [b]: measured by ICP-OES of the supernatant of reaction mixture of the dissolution step.

Table S6 Amount of recovered magnetic particles, leaching yields and isolated nanorattles yields for the dissolution reaction in order to form platinum nanorattles.

sample	recovered magnetic particles[a]	leaching yield[b]	isolated nanorattle yield (absolute)[c]
**5** (1 wt% Pt)	27.9%	30%	4.1%
**5** (20 wt% Pt)	51.5%	32%	25.1%

[a]: amount of magnetically separable particles determined gravimetrically, [b]: calculated from the cobalt content measured by ICP-OES (see Table S5), [c]: calculated from the wt% of cobalt and platinum in the sample (determined by elemental microanalysis) and the amount of non-dissolved, magnetically removed cobalt-platinum-alloy particles

5 Characterization of platinum nanorattles

5.1 Scanning (transmission) electron micrographs (STEM/SEM)

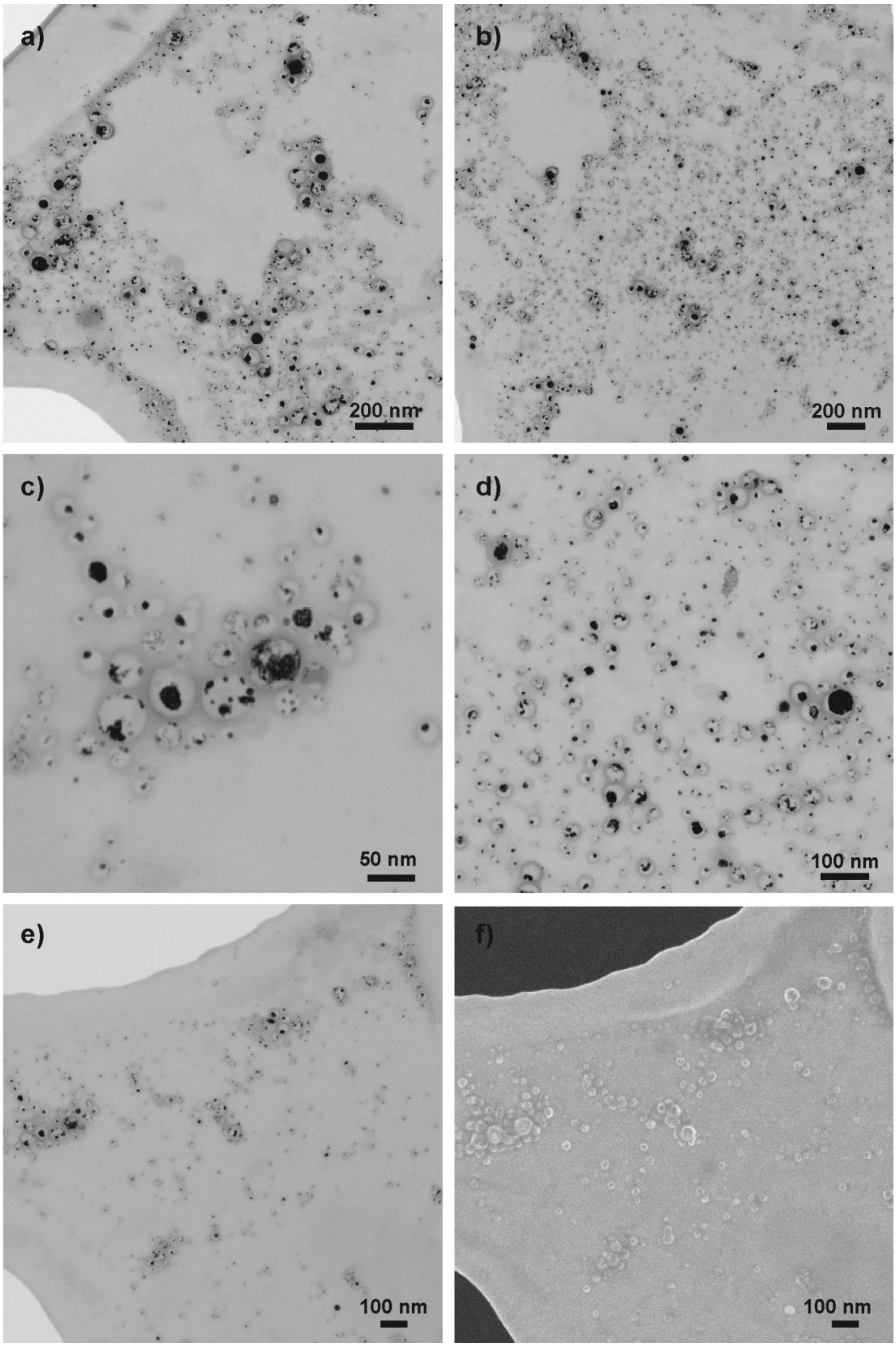

Fig. S6 Scanning transmission electron microscopy (STEM, a-e) and scanning electron microscopy (SEM, f) of platinum nanorattles made from carbon-coated cobalt-platinum-alloy nanoparticles (C/CoPt (20 wt% Pt)).

5.2 Transmission electron micrographs (TEM)

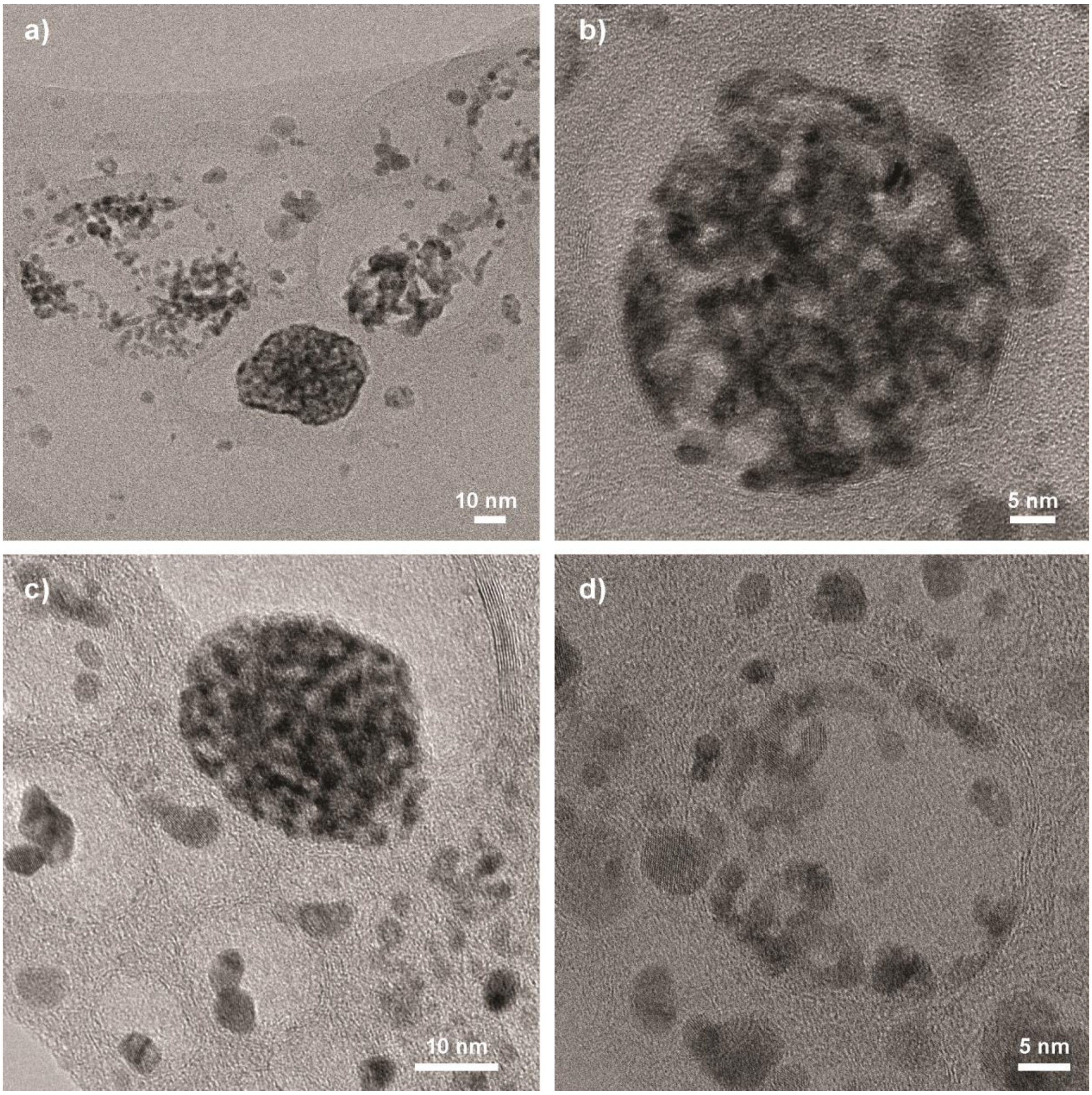

Fig. S7 Transmission electron micrographs of platinum nanorattles made from carbon-coated cobalt-platinum-alloy nanoparticles (C/CoPt (20 wt% Pt)).

5.3 Energy-dispersive X-ray spectroscopy (EDX)

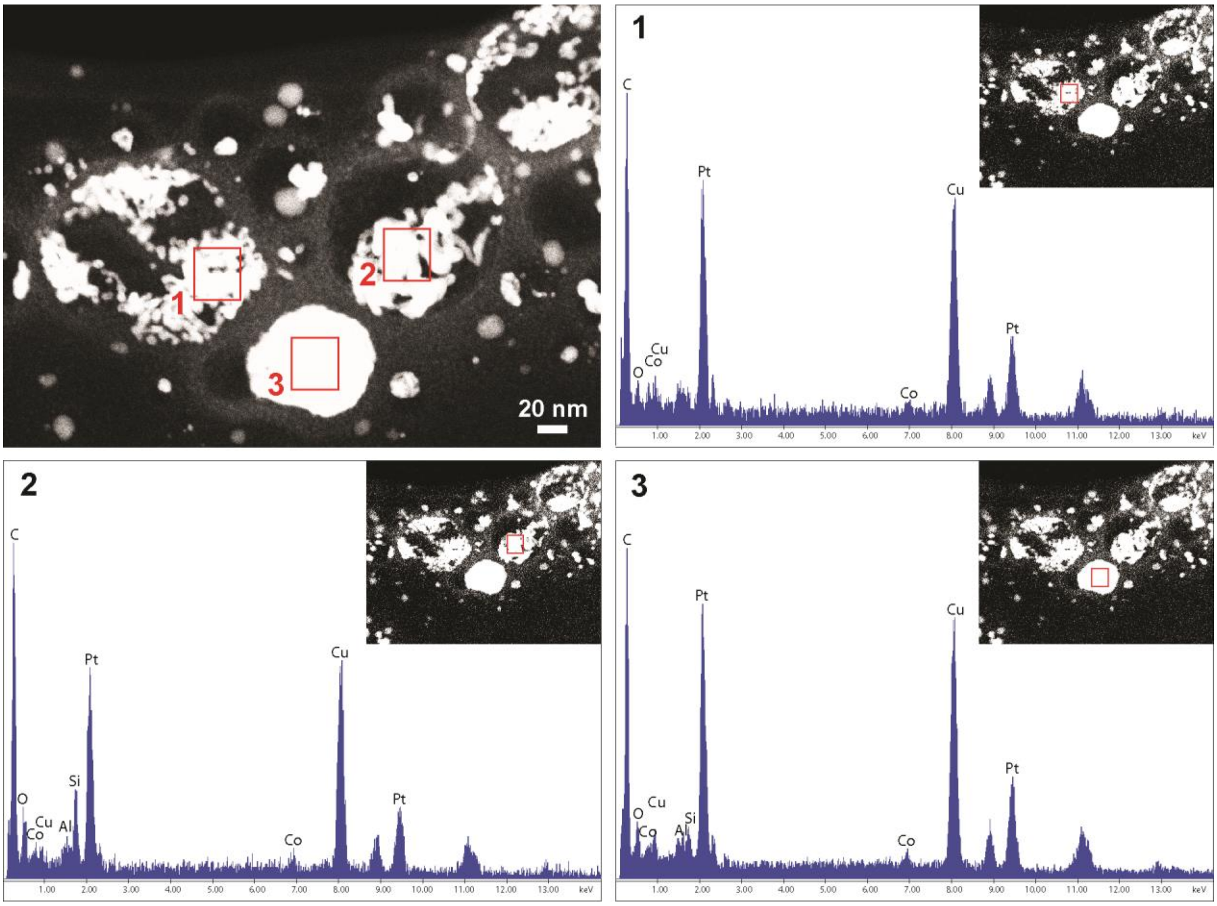

Fig. S8 Energy-dispersive X-ray spectroscopy (EDX) of platinum nanorattles made from C/CoPt (20 wt% Pt) nanoparticles.

5.4 Stereoscopic images

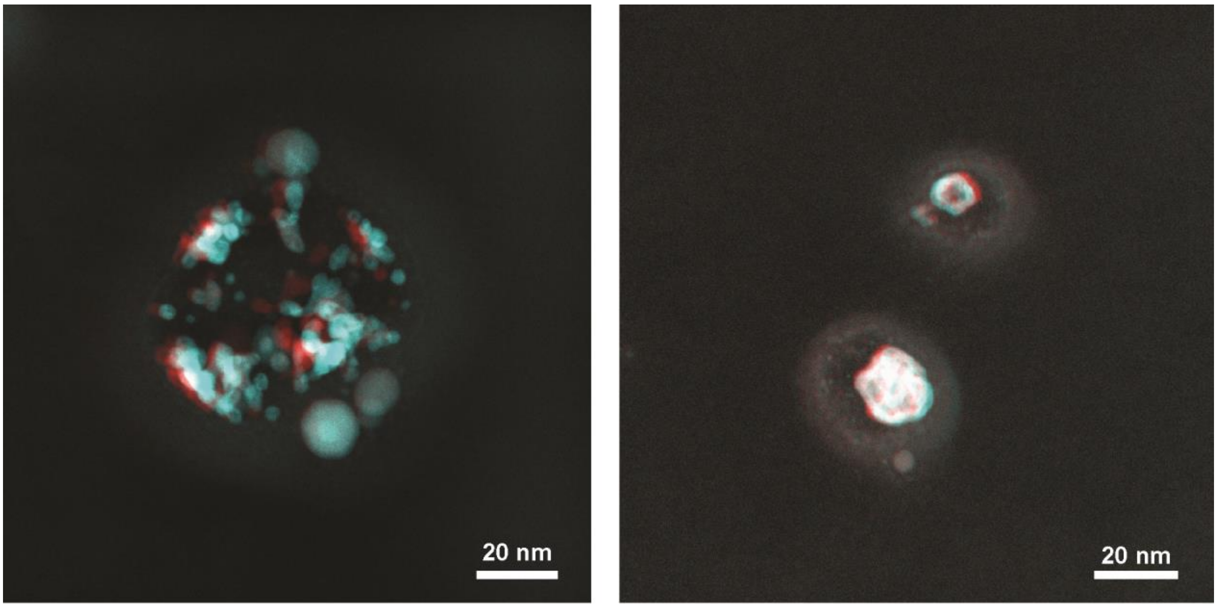

Fig. S9 Stereoscopic images of platinum nanorattles made from C/CoPt (20 wt% Pt) nanoparticles.

5.5 Scanning (transmission) electron micrographs (STEM/SEM)

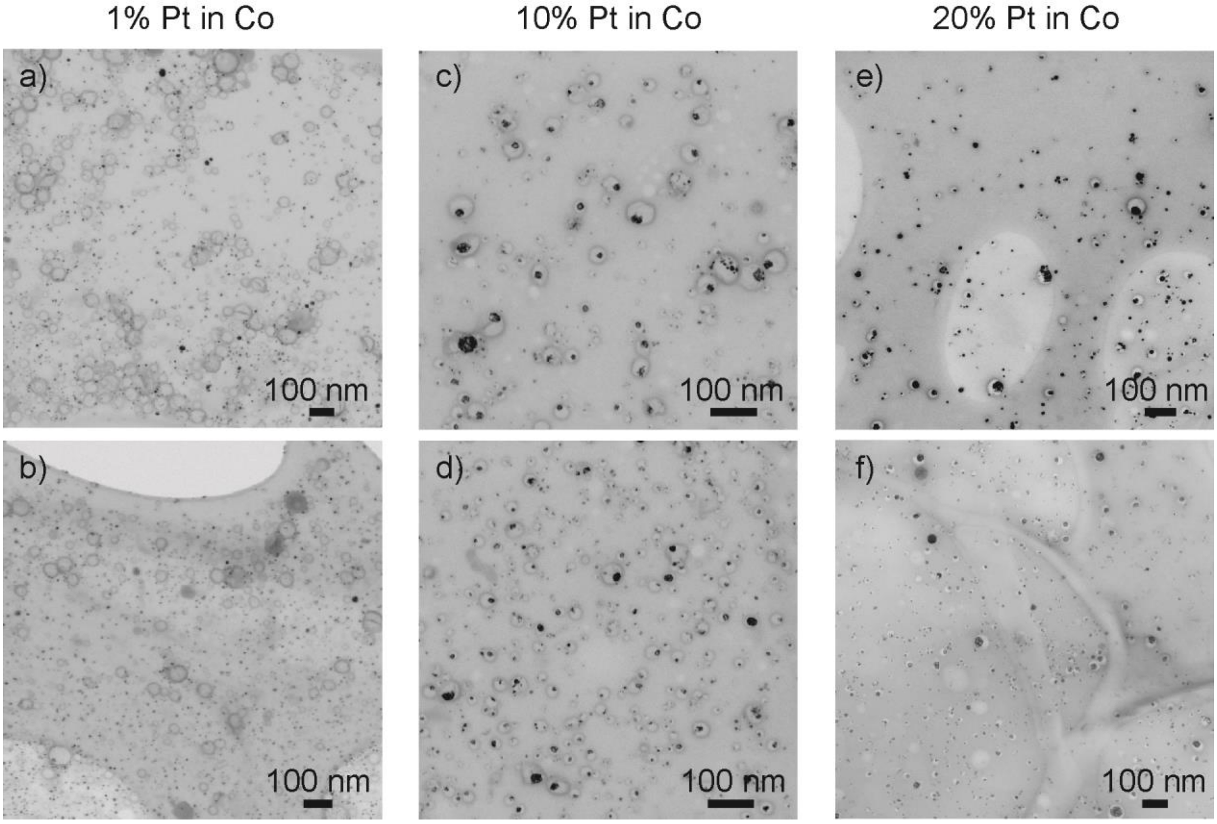

Fig. S 10 Scanning transmission electron microscopy of platinum nanorattles made from C/CoPt nanoparticles containing 1 wt% Pt (a, b), 10 wt% Pt (c, d) and 20 wt% Pt (e, f).

5.6 X-ray diffraction pattern (XRD)

Table S7 Particle diameters determined by peak width of XRD patterns using the Scherrer formula[4] and number- and volume-weighted mean particle diameters, determined by microscopy images, of platinum nanoparticles in the interior of the nanorattles and C/CoPt with different platinum contents (20 wt%, 10 wt% and 1 wt% platinum).

measurement method	Pt nanoparticles	C/CoPt (20 wt% Pt)	C/CoPt (10 wt% Pt)	C/CoPt (1 wt% Pt)
XRD	7.7 nm	13.6 nm	17.0 nm	16.4 nm
optical diameter analysis (number-weighted distribution)	3.8 nm	17.8 nm	19.5 nm	23.2 nm
optical diameter analysis (volume-weighted distribution)	5.3 nm	24.9 nm	27.6 nm	31.5 nm

Determining the crystal size from the XRD patterns for the C/CoPt (20 wt% Pt) nanoparticles resulted in a smaller size (13.6 nm) than the volume-weighted mean particle diameter determined by microscopy images (17.8 nm; see Table S7 for all particle diameters). This difference could be explained by the formation of polycrystalline particles or the formation of twins as previously observed for fcc-cobalt nanoparticles.[5]

5.7 Thermogravimetric analysis

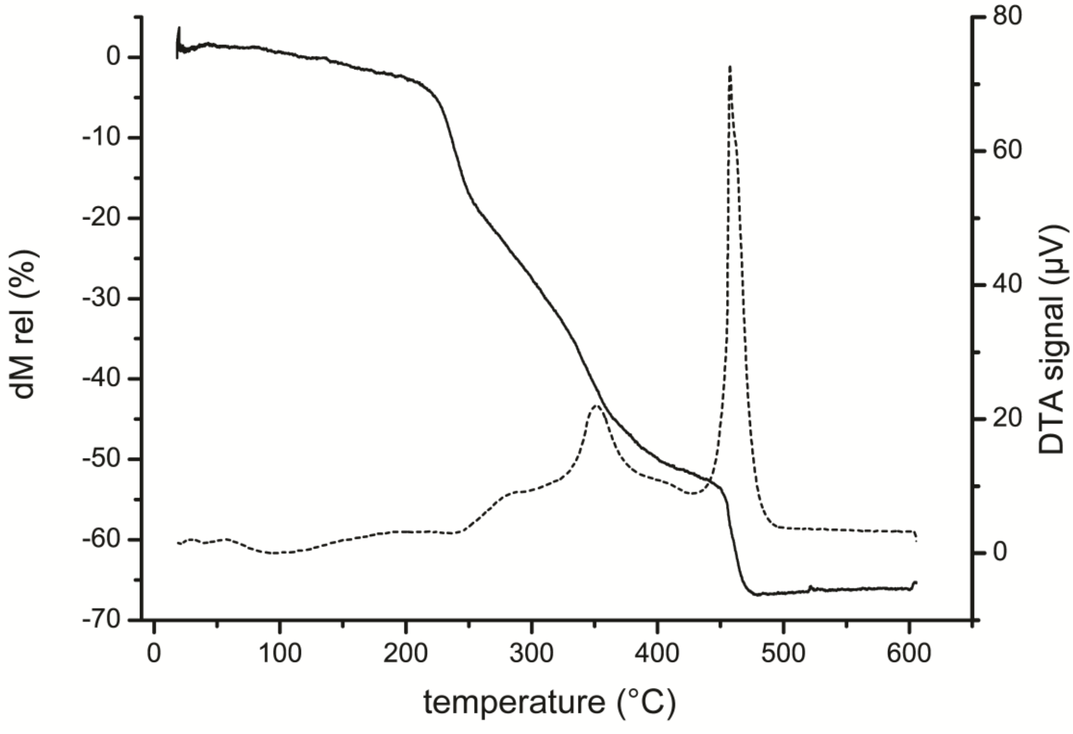

Fig. S11 Relative mass loss of Pt nanorattles derived from C/CoPt (20 wt% Pt) nanoparticles upon combustion of the organic part of the particles measured by thermogravimetry in air. Pt nanorattles showed the combustion of 66.9 wt% after heating to 600 °C.

5.8 Inductively coupled plasma time-of-flight mass spectrometry (ICP-TOFMS)

Table S8 Estimated and measured platinum and cobalt content of nanorattles made from C/CoPt (20 wt% Pt) nanoparticles and C/CoPt (1 wt% Pt) nanoparticles measured by ICP-TOFMS.

species	estimated Pt andCo content [a)]	measured Pt and Co content
Pt nanorattles<br>(20 wt% Pt)	Pt: 25.7 wt%<br>Co:0 wt%	Pt: 18.2 wt%<br>Co: 6.3 wt%
Pt nanorattles<br>(1 wt% Pt)	Pt: 2.0 wt%<br>Co: 0 wt%	Pt: 2.2 wt%<br>Co: 9.5 wt%

[a)]: calculated from the wt% of carbon of the C/CoPt nanoparticles, determined by elemental microanalysis, with the assumption of total cobalt dissolution and that the platinum content is the one which was aimed in the particle production process (meaning 20 wt% and 1 wt% platinum).

6 Size-selective catalysis

6.1 Catalytic oxidation of glucose and maltoheptaose by platinum catalysts

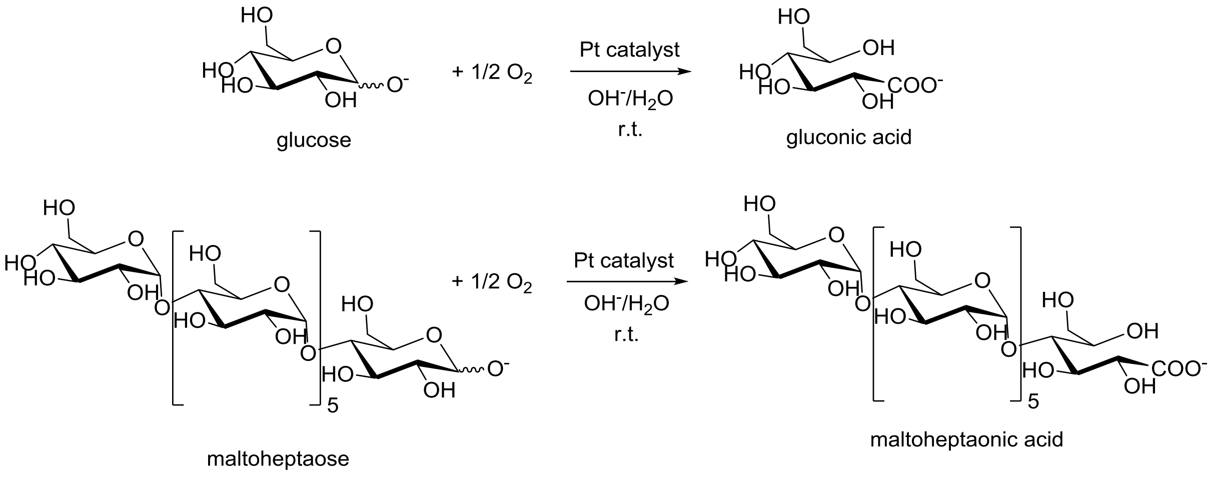

Scheme S2 Reaction equation of the platinum catalyzed oxidation of glucose and maltoheptaose to gluconic acid and maltoheptaonic acid, respectively.

Oxidation of glucose and maltoheptaose. 300 µL platinum catalyst (either (i) Pt/C (14 mg/10 mL H_2O, 70 ppm) or (ii) the same Pt/C solution 1 to 40 diluted (1.8 ppm) or (iii) platinum nanorattles (0.38 mg/mL, 70 ppm) or (iv) for the control no catalyst but only water) was added to a 2 mL Eppendorf tube. 37.5 µL KOH solution (3.33 M) was added. A solution containing glucose and maltoheptaose (each 6 mM in H_2O) was freshly prepared and 37.5 µL of it was added to the tube. It was shaken at room temperature and 1000 rpm. After 2 h, 5 h, 8 h and 24 h a sample (70 µL) was taken and filtered through a centrifugal filter unit (amicon ultra 0.5 mL, 14000 rcf, 20 minutes) to remove the catalyst. Instantly thereafter, mass spectrometry was performed.

As further control, the reaction was also performed with hollow carbon nanobubbles not containing platinum (0.19 mg/mL, HCNB[3]), carbon nanoparticles (0.38 mg/mL, nano activated carbon (100 nm), Nanostructured & Amorphous Materials, Inc.) and Pt nanoparticles made in the presence of hollow carbon nanobubbles (see Figure S14 for STEM images and below for synthesis procedure).

All signal of the mass spectrometry measurements were normalized by the corresponding control (case iv in the above description).

6.2 Scanning transmission electron micrographs (STEM) of Pt nanorattles after usage as catalyst

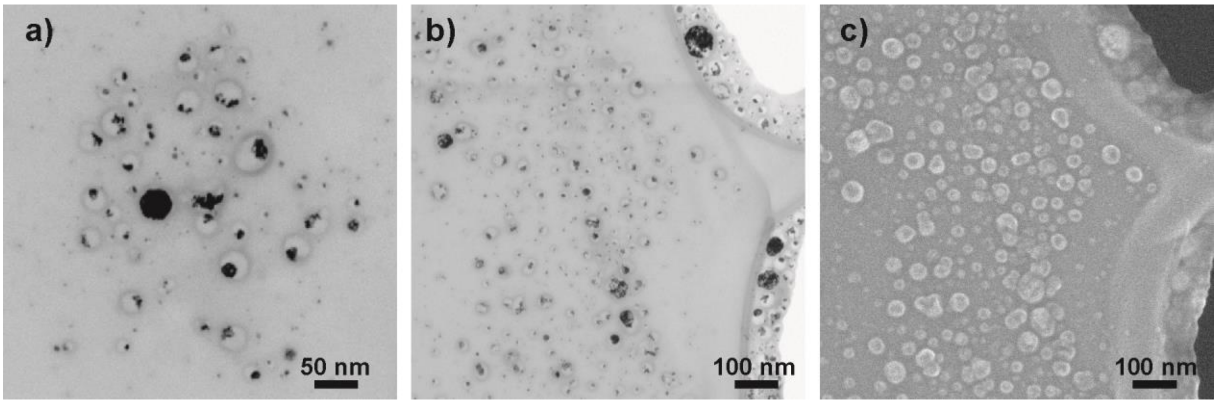

Fig. S12 Scanning transmission electron microscopy (STEM, a, b) and scanning electron microscopy (SEM, c) of platinum nanorattles made from C/CoPt (20 wt% Pt) nanoparticles after usage as catalyst for the oxidation of glucose and maltoheptaose.

6.3 Results of control reactions

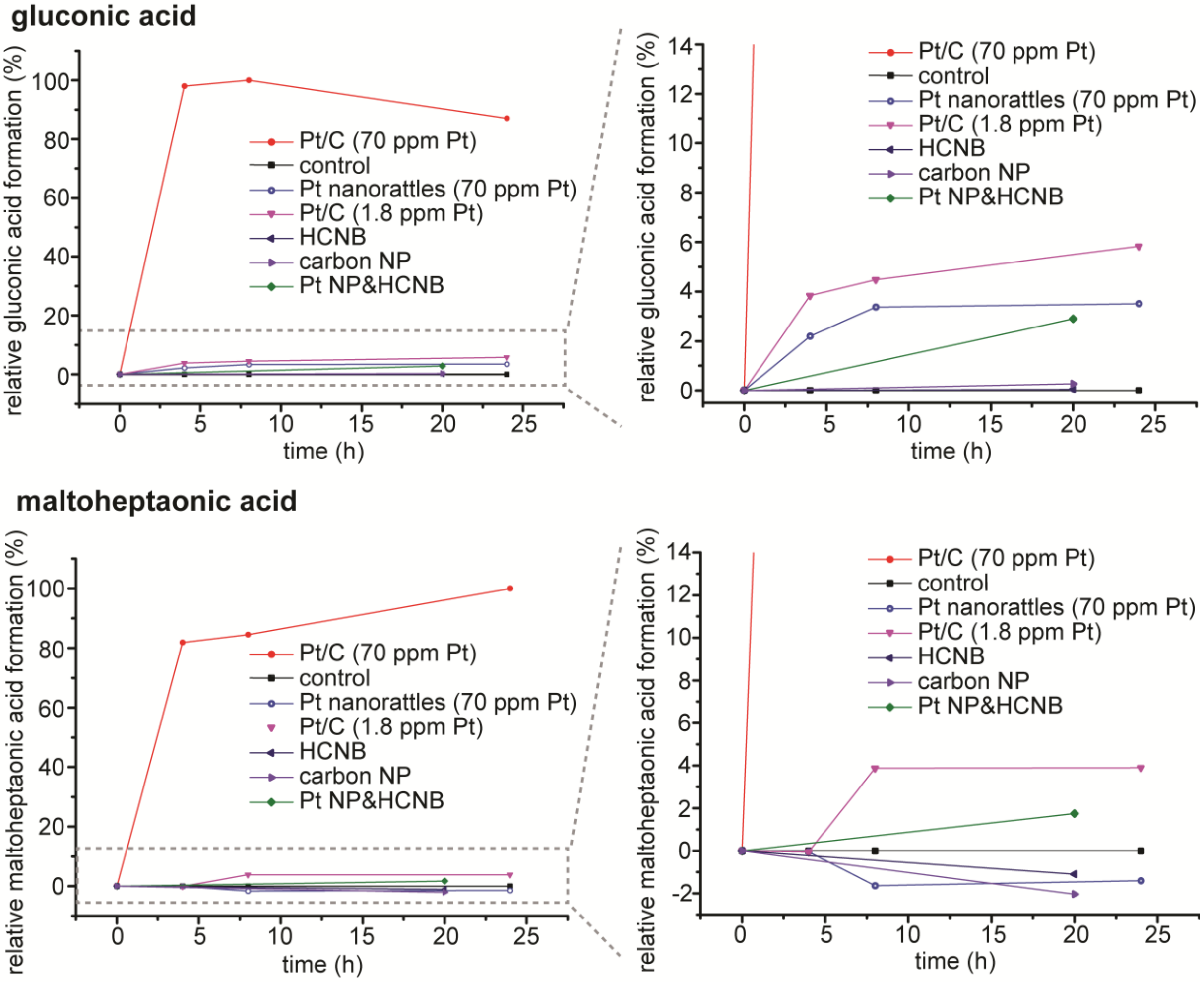

Fig. S13 Results of control reactions for the platinum catalyzed oxidation of glucose and maltoheptaose with hollow carbon nanobubbles (HCNB[3]; empty nanorattles), spherical carbon nanoparticles and platinum nanoparticles, which were made by reduction in the presence of empty nanorattles (Pt NP & HCNB; see Figure S14).

6.4 Formation of platinum nanoparticles in the presence of nanobubbles (Pt NP & HCNB)

Synthesis of Pt NP & HCNB. The procedure was adapted from literature[6]. An aqueous solution of H_2PtCl_6 (0.25 mL, 0.172 mM) was added to an aqueous solution of hollow carbon nanobubbles (synthesized as described before[3], 0.25 mL, 0.19 mg bubbles / mL). Then, $NaBH_4$ (0.5 mL, 5 mM) was added dropwise. The reaction was shaken for 1 h at 500 rpm and then 1 h at 1000 rpm at room temperature. The nanobubbles were washed by filtration (amicon ultra centrifugal filter 4 mL, 50 kDa).

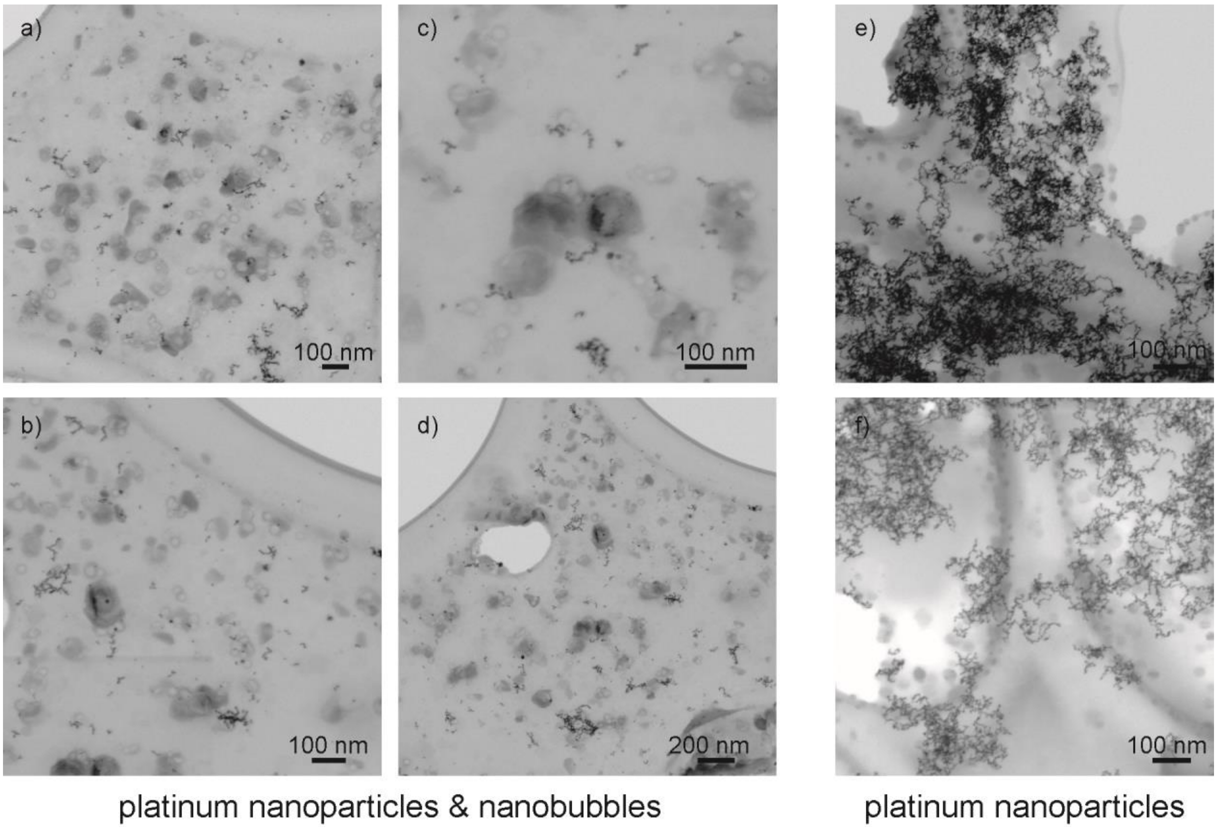

Fig. S14 Scanning transmission electron microscopy of platinum nanoparticles made in the presence of hollow carbon nanobubbles (a-d) and platinum nanoparticles made without the presence of hollow carbon nanobubbles as reference (e, f).

7 References

1. R. N. Grass, E. K. Athanassiou and W. J. Stark, *Angew. Chem. Int. Ed.*, 2007, **46**, 4909-4912.
2. C. J. Hofer, V. Zlateski, P. R. Stoessel, D. Paunescu, E. M. Schneider, R. N. Grass, M. Zeltner and W. J. Stark, *Chem. Commun.*, 2015, **51**, 1826-1829.
3. C. J. Hofer, R. N. Grass, M. Zeltner, C. A. Mora, F. Krumeich and W. J. Stark, *Angew. Chem. Int. Ed.*, 2016, **55**, 8761-8765.
4. H. Borchert, E. V. Shevchenko, A. Robert, I. Mekis, A. Kornowski, G. Grübel and H. Weller, *Langmuir*, 2005, **21**, 1931-1936.
5. R. N. Grass and W. J. Stark, *J. Mater. Chem.*, 2006, **16**, 1825-1830.
6. S. Wu, Z. Zhu, T. Siepenkötter and X. Guo, *Asia-Pac. J. Chem. Eng.*, 2012, **7**, 886-891.
